# Otofaciocervical Syndrome and Its Overlap with Branchiootorenal Spectrum: An Integrated Literature Analysis of *EYA1*-Related Disorders, Including a Novel Case with an 8q13.2q13.3 Deletion

**DOI:** 10.3390/genes16111267

**Published:** 2025-10-28

**Authors:** Ludovico Graziani, Miriam Lucia Carriero, Salvatore Melchionda, Bartolomeo Augello, Orazio Palumbo, Mario Bengala, Marco Castori, Giuseppe Novelli

**Affiliations:** 1 Department of Biomedicine and Prevention, University of Rome “Tor Vergata”, 00133 Rome, Italy; 2 Genetics and Developmental Biology Unit, Azienda Ospedaliera Universitaria Sassari, 07100 Sassari, Italy; 3 UOC Genetica Medica, Fondazione IRCCS Casa Sollievo della Sofferenza, 71013 San Giovanni Rotondo, Italyo.palumbo@operapadrepio.it (O.P.);; 4 Medical Genetics Unit, “Tor Vergata” University Hospital, 00133 Rome, Italy

**Keywords:** *EYA1*, BORSD, OTFCS, genotype–phenotype correlations, allelic disorders, 8q13.3

## Abstract

Otofaciocervical syndrome (OTFCS) is a rare disorder characterized by facial, auditory, and shoulder girdle anomalies. Its significant phenotypic overlap with branchiootorenal spectrum disorders (BORSD)—both linked to *EYA1* (EYA transcriptional coactivator and phosphatase 1) gene defects—has raised questions about whether they are distinct entities or part of a single clinical spectrum. We report a novel OTFCS patient with a de novo microdeletion spanning *EYA1* and review all published cases of *EYA1*-related disorders. Our analysis reveals that all *EYA1* variant types (truncating, missense, CNV, etc.) can cause BORSD, OTFCS, or hybrid phenotypes, firmly supporting their status as allelic disorders. Crucially, all reported OTFCS patients with *EYA1* variants had renal anomalies, a feature previously considered a hallmark of BORSD. We conclude that BORSD and OTFCS constitute a single *EYA1*-related diagnostic continuum. This reclassification mandates the development of follow-up protocols that integrate renal, otologic, and skeletal surveillance in *EYA1*-related disorders, including OTFCS, and refines prognostic and genetic counseling.

## 1. Introduction

Craniofacial syndromes associated with branchial arch anomalies represent a clinically and genetically heterogeneous group of disorders, often characterized by overlapping features that complicate diagnosis and etiological classification [1,2]. Among these, Otofaciocervical syndrome (OTFCS) is a rare genetic disorder first described by Fara et al. in 1967, with fewer than ten cases reported in the literature [3,4]. It is characterized by peculiar craniofacial traits (e.g., long triangular face, broad forehead, narrow nose and mandible, and high arched palate), ear abnormalities (e.g., low-set, cup-shaped ears with prominent conchae and a hypoplastic tragus and lobe) often associated with hearing loss, and shoulder girdle anomalies (sloping shoulders, low-set clavicles, winged scapulae, and trapezius hypoplasia). Skeletal anomalies other than girdle anomalies and nasolacrimal duct defects are frequently reported, whereas neurodevelopmental delay and short stature are observed only in some patients [5,6]. OTFCS shares significant phenotypic overlap with branchiootorenal spectrum disorders (BORSD) [7,8]. Nonetheless, they have been previously described as clinically distinct entities: phenotypic traits such as facial dysmorphisms and shoulder girdle anomalies were considered specific to OTFCS, whereas BORSD were explicitly characterized by functional and structural renal anomalies (Table 1) [9,10].

Heterozygous variants in *EYA1* (EYA transcriptional coactivator and phosphatase 1) account for approximately 40–75% of individuals clinically diagnosed with BORSD [11,12], but have also been reported in OTFCS patients [13,14,15]. Other genes in the Pax-Six-Eya-Dach network (PSEDN) are likewise implicated in both phenotypes. Heterozygous variants in *SIX1* (sine oculis homeobox homolog 1) and *SIX5* (sine oculis homeobox homolog 5) have been detected in 3.0–45% and 0–3.1% of individuals with BORSD, respectively [16,17,18]. In addition, biallelic *PAX1* (paired box 1) variants underlie OTFCS type 2 with T-cell deficiency (OTFCS2) [19,20,21,22], while loss-of-function (LoF) variants in *EYA4* (EYA transcriptional coactivator and phosphatase 4) have more recently been reported in a single affected family [4].

Whether OTFCS and BORSD represent distinct nosological entities or instead form part of a broader phenotypic continuum remains unresolved, as the precise genetic basis of OTFCS is not yet fully clarified. Importantly, some individuals with a BORSD diagnosis present with features typical of OTFCS—musculoskeletal and neurodevelopmental involvement—while some OTFCS patients exhibit renal anomalies, suggesting that the two conditions may, at least in part, represent allelic disorders [11,23,24]. This growing body of evidence supports the hypothesis that these disorders may, at least in part, represent allelic conditions.

In this study, we report a novel patient presenting with OTFCS harboring a de novo microdeletion encompassing *EYA1* and perform a comprehensive review of all published cases of *EYA1*-related disorders. By delineating overlaps and distinctions between OTFCS and BORSD, we aim to refine their allelic relationship, improve diagnostic precision, and inform genetic counseling, while contributing to a deeper understanding of the molecular mechanisms underlying these syndromes.

## 2. Materials and Methods

### 2.1. Clinical and Molecular Data

Clinical and audiological information was collected for the index patient, including detailed phenotypic characterization with particular attention to branchial, auricular, renal, and neurodevelopmental features. Audiological assessments included the type and degree of hearing loss. Genomic DNA was extracted from peripheral blood samples.

Chromosome microarray analysis (CMA) was performed using the CytoScan XON array (Thermo Fisher Scientific, Waltham, MA, USA).

Multiplex Ligation-dependent Probe Amplification (MLPA) was performed using the SALSA MLPA probemix P153-B1 *EYA1* kit (MRC-Holland, Amsterdam, The Netherlands), and variant analysis was carried out with Coffalyser.Net software v.240129.1959 (MRC-Holland, Amterdam, The Netherlands). The coordinates of detected deletions were mapped to the human genome assembly hg38 (GRCh38). Segregation analysis was performed to determine the inheritance pattern.

### 2.2. Literature Review and Data Extraction

A systematic literature review was conducted (last search: August 2025) using PubMed, Scopus, Embase, and Google Scholar, with the following keywords: “BORSD”, “BOR syndrome”, “BO syndrome”, “OFC syndrome”, “OTFC syndrome”, “BOF syndrome”, “BOU syndrome”, “branchio-oto-renal”, “branchio-otic”, “Otofaciocervical”, “del”, “deletion”, and “*EYA1*”. Filters applied: English language, human studies, and original clinical/genetic data.

### 2.3. Inclusion and Exclusion Criteria

Included: case reports, series, or cohorts with (i) *EYA1* SNVs/indels or CNVs and (ii) patient-level clinical data covering ≥2 domains (branchial, otologic, renal, craniofacial, musculoskeletal). Excluded: reviews, functional-only/animal studies, or overlapping cohorts (retaining the most complete report).

### 2.4. Screening and Data Extraction

Two reviewers independently screened titles/abstracts, followed by a full-text review. Extracted data included demographics, clinical features (categorized as BORSD-typical or OTFCS-typical), variant type (missense, truncating, splice, indel, stop-loss, structural/CNV), and deletion coordinates. All variants were described according to HGVS nomenclature using the *EYA1* transcript NM_000503.6 and mapped to GRCh38. Duplicates were removed.

## 3. Clinical Presentation

**Figure 1:**
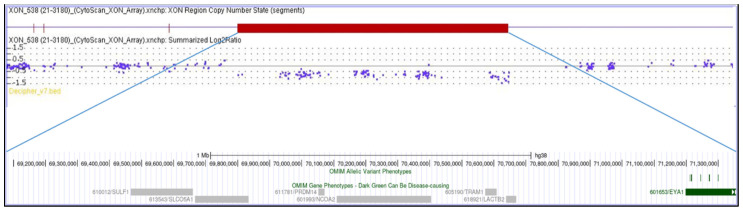
The molecular karyotype of the novel patient and the associated genes, adapted from the UCSC Genome Browser according to the International System for Human Cytogenetic Nomenclature (ISCN 2024): arr[GRCh38] 8q13.2q13.3(69,068,130_71,362,732)×1.

A 22-year-old female, born at term, second child of healthy non-consanguineous parents, presented with severe congenital bilateral mixed hearing loss, bilateral preauricular fistulas, hypoplasia of the left shoulder muscles, winged scapula, short stature (<3rd percentile), and a history of speech delay. Chromosome analysis revealed a normal female karyotype (46,XX). Analysis of the *EYA1* gene was negative for variants using sequencing approaches. MLPA analysis identified a heterozygous de novo deletion encompassing the entire coding region of *EYA1* at 8q13.3. CMA (Figure 1) confirmed a 2.3 Mb interstitial deletion at 8q13.2q13.3 chromosomal region, which spanned from nucleotides 69,068,130 to 71,362,732 (GRCh38) and involved 12 genes (*LINC01592*, *LINC01603*, *SULF1*, *SLCO5A1*, *PRDM14*, *NCOA2*, *LOC101926892*, *TRAM1*, *LACTB2-AS1*, *LACTB2*, *XKR9*, EYA1), and which are further characterized in Table 2. The microdeletion occurred de novo because both parents were wild-type.

## 4. Results

**Figure 2:**
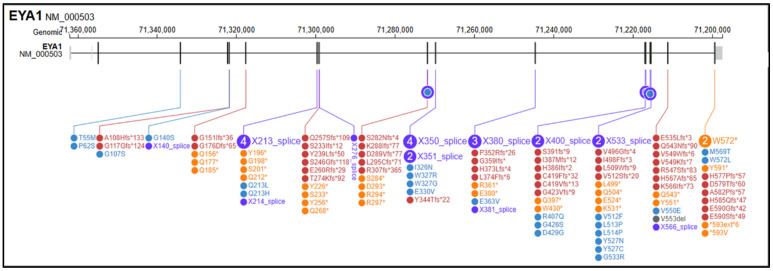
Graphical representation of variants in the *EYA1* (NM_000503.6) gene reported in medical and scientific literature (PubMed, Scopus, Google Scholar) in association with BORSD/OTFCS phenotypes. In yellow, frameshift variants; in purple, splicing variants; in red, nonsense variants; in blue, missense variants; in black, stop-loss variants; in grey, in/del variants. Variant visualization was generated using ProteinPaint Release version: 2.147.0-ecd631c6f (St. Jude Children’s Research Hospital, Memphis, TN, USA; https://proteinpaint.stjude.org (accessed on 25 September 2025).

The search retrieved more than 200 records in PubMed and additional records in Scopus, Embase and Google Scholar; after deduplication and eligibility screening, 55 studies and 141 reported SNVs were included, as described in Supplementary Materials. Among these, 54 (38.3%) were frameshift variants (fs), 30 (21.3%) were nonsense variants (ns), 28 (19.9%) were splice-site variants (sp), 26 (18.4%) were missense variants (ms), 2 (1.4%) were stop-loss/stop-like variants (sl), and 1 (0.7%) was annotated as an indel (Figure 2). *EYA1* gene SNVs found in the literature in association with OTFCS/BORSD spectrum are shown in Table 3, according to the first accession of genotype and/or complete phenotype. 1 splice site variant was associated with a single OTFCS case, 1 missense variant was associated with unrelated OTFCS and BORSD cases, while all the remainder SNVs were detected within the BORSD spectrum. Renal involvement is apparently absent in association with 34/141 (24.1%) described SNVs, regardless of the type of variant (ns, fs, sp, ms, sl) within the BORSD spectrum. OTFCS cases (both due to SNVs and CNVs) are further characterized in Table 4 and compared to the reported case.

In addition to the distribution of variant classes, our analysis revealed that large *EYA1* deletions are enriched among BORSD cases, accounting for approximately 20% of the reports in the literature. Moreover, two-thirds of reported *EYA1* SNVs were predicted to be LoF, consistent with haploinsufficiency as the main disease mechanism.

## 5. Discussion

The present review highlights the complex relationship between BORSD and OTFCS, both associated with *EYA1* copy number and sequence variants. BORSD has traditionally been defined by a triad of branchial, otologic, and renal anomalies [7,18]. In contrast, OTFCS has been described as a distinct condition, characterized by musculoskeletal anomalies such as scapular dysplasia and short stature [9,14]. However, our systematic analysis and the present case emphasize that considerable phenotypic overlap exists, and that classical BORSD features may co-occur with OTFCS hallmarks.

The EYA proteins are components of a conserved regulatory network that is often referred to as the PAX–SIX–EYA–DACH developmental network (PSEDN) to better reflect the proteins involved [69]. This network plays a key regulatory role in the early development of multiple organs [70,71]. Notably, all known disease genes implicated in BORSD and OTFCS belong to this network. While OTFCS has also been genetically linked to *PAX1* [4,14] and, in a limited number of patients, *EYA4* in [19], *EYA1* remains the major gene implicated in conditions.

Pathogenic *EYA1* variants encompass truncating, missense, splice-site, stop-loss, and copy-number alterations, and have been documented in association with BORSD, OTFCS, and intermediate phenotypes [6,62]. Within our case series, one SNV in the *EYA1* gene was described exclusively in association with OTFCS (1/141; 0.7%), one SNV was described in association with both OTFCS and BORSD (1/141; 0.7%), while the remainder (139/141; 98.6%) were described within the BORSD spectrum, with or without renal involvement. Thus, the variant class alone may be insufficient to predict the clinical presentation. This supports the view that haploinsufficiency is the predominant disease mechanism [72,73], but additional genetic or environmental modifiers likely influence phenotypic expressivity. Importantly, the observation that OTFCS can also result from missense and splice variants, and not exclusively from large deletions, further challenges the concept of OTFCS as a purely contiguous gene deletion syndrome [9,14]. Complex rearrangements, inversions, and insertions further contribute to the mutational spectrum [74,75].

A particularly noteworthy finding from our review is that the majority of published patients with OTFCS due to *EYA1* defects presented with renal anomalies, while in approximately 25% of *EYA1*-related cases of BORSD, they were absent regardless of the variant type. Since renal involvement has been traditionally associated with BORSD, this observation undermines the concept of a strict clinical separation between the two syndromes. Instead, it suggests that musculoskeletal involvement in OTFCS and renal anomalies in BORSD are not mutually exclusive, but somewhat variable manifestations of the same allelic defect. Conversely, OTFCS caused by other genetic mechanisms may represent distinct subtypes, supporting the concept of a subclassification of OTFCS according to the underlying molecular etiology.

To our knowledge, this is the first reported case of OTFCS due to an *EYA1* defect characterized by Chromosomal Microarray (CMA). This provides a precise genomic definition of the 8q13.2q13.3 microdeletion underlying the phenotype and allows a detailed assessment of co-deleted genes. We also acknowledge that the interpretation of microdeletion cases is complicated by the involvement of multiple genes. In our case, the 8q13.2q13.3 deletion spans additional genes besides *EYA1*. We therefore reviewed their known or predicted functions. Although some have roles in developmental pathways, none have been consistently implicated in renal or musculoskeletal phenotypes resembling BORSD or OTFCS. Thus, while we cannot completely rule out modifying effects from neighboring genes, the current evidence supports *EYA1* haploinsufficiency as the principal driver of both the craniofacial–musculoskeletal anomalies and the renal phenotype in our patient.

The wide spectrum of presentations of *EYA1*-related disorders suggests that modifying factors, such as genetic background, environmental influences, or stochastic events during development, may critically modulate the expressivity of *EYA1* variants [76]. Analogous patterns are well recognized in other genetic conditions such as *COL2A1* (Collagen, Type II, Alpha-1)-related skeletal dysplasias and *TBX6* (T-Box Transcription Factor 6)-related segmentation defects, where allelic heterogeneity and modifiers account for wide phenotypic variability [77,78,79,80]. Rather than being distinct syndromes, BORSD and OTFCS may represent different clinical expressions of *EYA1* dysfunction within the context of the broader PSEDN. Reports of identical or highly similar *EYA1* anomalies resulting in divergent phenotypes in different families further support this model [35,72,81].

From a clinical standpoint, acknowledging OTFCS and BORSD as allelic disorders has significant implications. It underscores the need to systematically evaluate musculoskeletal and developmental features in patients diagnosed with BORSD, and conversely, to ensure comprehensive renal and auditory assessments in patients with OTFCS. Grouping both under the umbrella of *EYA1*-related disorders would enhance and streamline variant interpretation, strengthen genetic counseling, and support the development of follow-up protocols that integrate renal, otologic, and skeletal surveillance.

Future studies should pursue three main directions: (i) large-scale genotype–phenotype analyses integrating both BORSD and OTFCS cases; (ii) functional studies to elucidate the molecular impact of different *EYA1* variants; and (iii) investigation of potential second-site modifiers within the PSEDN Network that might influence phenotypic outcome.

## 6. Conclusions

Our findings consolidate the model of BORSD and OTFCS as allelic disorders within a unified *EYA1*-related spectrum. This reclassification is critical for clinical practice: it improves diagnostic accuracy, mandates comprehensive phenotyping—most notably, systematic renal screening in all OTFCS patients—and refines prognostic and genetic counseling. Future research integrating deep phenotyping, genomic data, and functional studies will be essential to elucidate the mechanisms underlying the striking phenotypic variability within this spectrum.

## Figures and Tables

**Table 1:** Genotypic and phenotypic overlapping within the branchiootorenal and Otofaciocervical syndrome spectrum.

Disorder		Genotype			Phenotype						
		Gene	OMIM	Inher.	Branchial	Ear	Renal	Eye	Musculoskeletal	Neurologic	Immunologic
BORS											
	Type 1	*EYA1*	113650	AD	+	+	+	±	−	−	−
	Type 2	*SIX5*	610896	AD	+	+	+	−	−	−	−
BOS											
	Type 1	*EYA1*	120502	AD	+	+	−	±	−	−	−
	Type 2	-	602588	AD	+	+	−	−	−	−	−
	Type 3	*SIX1*	608389	AD	+	+	−	−	−	−	−
OTFCS											
	Type 1	*EYA1*	166780	AD	+	+	+	−	+	+	±
	Type 2	*PAX1*	615560	AR	+	+	−	±	+	+	+

BORS, Branchiootorenal syndrome; BOS, Branchiootic syndrome; OTFCS, Otofaciocervical syndrome; AD, Autosomal dominant; AR, Autosomal recessive; OMIM, Online Mendelian Inheritance in Man; +, present; −, absent; ± variable.

**Table 2:** OMIM genes within the patient’s deleted chromosomal region. pHaplo dosage sensitivity index is presented, where high ranks (i.e., 0.8–1.0) indicate a gene is more likely to exhibit haploinsufficiency and low ranks (i.e., 0.0–0.20) indicate a gene is more likely not to exhibit haploinsufficiency.

Gene	MIM Number	pHaplo	Phenotypes	Notes
*EYA1*	601653	0.90	BORS, OTFCS.	-
*LACTB2*	618921	0.31	-	May have a role in mitochondrial function and cell viability (Yu et al., 2016) [25]
*NCOA2*	601993	0.99	-	Encodes a nuclear receptor coactivator, which aids in the function of nuclear hormone receptors (Cai et al., 2019) [26]
*PRDM14*	611781	0.59	-	Gene amplification has frequently been observed in human tumors (Nishikawa et al., 2007) [27]
*SLCO5A1*	613543	0.35	-	Highly expressed in fetal and adult brain and heart (Isidor et al., 2010) [28]
*SULF1*	610012	0.66	-	Involved in cell signaling by heparin-binding growth factors (Lai et al., 2003) [29]
*TRAM1*	605190	0.88	-	Functional analysis indicated that it influences glycosylation and is stimulatory or required for the translocation of secretory proteins (Gorlich et al., 1992) [30]

BORS, Branchiootorenal syndrome; AD, Autosomal dominant; OTFCS, Otofaciocervical syndrome; OMIM, Online Mendelian Inheritance in Man.

**Table 3:** *EYA1* variants have been reported in patients with branchiootorenal spectrum disorders (BORSD), branchiootic (BO) syndrome, or otofaciocervical syndrome (OTFCS). Variants are described according to the HGVS nomenclature, using the reference transcript NM_000503.6 (*EYA1*) and mapped to the human genome assembly GRCh38. Variant types are classified as missense (ms), nonsense (ns), frameshift (fs), splice (sp), insertion/deletion (indel), or stoploss (sl). Clinical diagnoses are reported as indicated in the original publications, grouped into BOR, BO, OTFCS, or overlapping phenotypes. Only molecularly confirmed cases with sufficient clinical description were included. References correspond to the first report of each genotype–phenotype association.

Genotype				Phenotype	Reference
CDS (c.)	Protein (p.)	Exon(s)	Variant type		Author
164C>T	Thr55Met	4	ms	BOR	Orten et al., Hum. Mutat. (2008) [31]
283C>T	Pro62Ser	6	ms	BOR	Krug et al., Hum. Mutat. (2011) [11]
321del	Ala108HisfsTer133	6	fs	BOR	Lee et al., Ann. Clin. Lab Sci. (2009) [32]
348del	Gly117GlufsTer124	6	fs	BOR	Orten et al., Hum. Mutat. (2008) [31]
402C>A	Gly107Ser	6	ms	BOR	Orten et al., Hum. Mutat. (2008) [31]
418G>A	Gly140Ser	6	ms	BOR/BO	Krug et al., Hum. Mutat. (2011), Kim et al., Mol. Biol. Rep. (2014) [11,33]
418+1G>C	Invariant ‘gt’	IVS6	sp	BOR	Unzaki et al., J. Hum. Genet. (2018) [24]
450_451del	Gly151IlefsTer36	7	fs	BOR	Orten et al., Hum. Mutat. (2008) [31]
466C>T	Gln156Ter	7	ns	BOR	Wang et al., Laryngoscope (2012) [34]
525del	Gly176AspfsTer65	7	fs	BOR	Klingbeil et al., Int. J. Pediatr. Otorhinolaryngol. (2017) [35]
529C>T	Gln177Ter	7	ns	BOR	Krug et al., Hum. Mutat. (2011) [11]
553C>T	Gln185Ter	7	ns	BOR	Orten et al., Hum. Mutat. (2008) [31]
588T>G	Tyr196Ter	8	ns	BO	Ideura et al., Sci. Rep. (2019) [36]
592G>T	Gly198Ter	8	ns	BOR	Orten et al., Hum. Mutat. (2008) [31]
602C>G	Ser201Ter	8	ns	BO	Orten et al., Hum. Mutat. (2008) [31]
634C>T	Gln212Ter	8	ns	BOR	Orten et al., Hum. Mutat. (2008) [31]
638A>T	Gln213Leu	8	ms	BOR	Orten et al., Hum. Mutat. (2008) [31]
639G>C	Gln213His	8	ms	BOR	Orten et al., Hum. Mutat. (2008) [31]
639+1G>A	Invariant ‘gt’	IVS8	sp	OTFC	Estefanía et al., Ann. Hum. Genet. (2006) [13]
639+1G>C	Invariant ‘gt’	IVS8	sp	BOR	Orten et al., Hum. Mutat. (2008) [31]
639+2del	Invariant ‘gt’	IVS8	sp	BOR	Orten et al., Hum. Mutat. (2008) [31]
639+3A>C	exon skipping	IVS8	sp	BOR	Zhang et al., BMC Med. Genomics (2024) [37]
640-15G>A	New splice acceptor	IVS8	sp	BOR	Orten et al., Hum. Mutat. (2008) [31]
769del	Gln257SerfsTer109	9	fs	BOR	Krug et al., Hum. Mutat. (2011) [11]
678C>A	Tyr226Ter	9	ns	BOR	Riedhammer et al., Eur. J. Hum. Genet. (2023) [38]
685_695dup	Ser233IlefsTer12	9	fs	BOR	Krug et al., Hum. Mutat. (2011) [11]
698C>A	Ser233Ter	9	ns	BOR	Unzaki et al., J. Hum. Genet. (2018) [24]
715dup	Tyr239LeufsTer50	9	fs	BOR	Krug et al., Hum. Mutat. (2011) [11]
735_743delCAGCCCAACinsTG	Ser246GlyfsTer118	9	fs	BOR	Krug et al., Hum. Mutat. (2011) [11]
768C>A	Tyr256Ter	9	ns	BO	Orten et al., Hum. Mutat. (2008) [31]
777dup	Glu260ArgfsTer29	9	fs	BOR	Orten et al., Hum. Mutat. (2008) [31]
802C>T	Gln268Ter	9	ns	BOR	Cho et al., Int. J. Mol. Sci. (2024) [8]
821del	Thr274LysfsTer92	9	fs	BOR	Krug et al., Hum. Mutat. (2011) [11]
827-1G>C	Invariant ‘at’	IVS9	sp	BOR	Tang et al., Medicine (Baltimore) (2022) [39]
845_852del	Ser282AsnfsTer4	10	fs	BOR	Orten et al., Hum. Mutat. (2008) [31]
851C>G	Ser284Ter	10	ns	BOR	Orten et al., Hum. Mutat. (2008) [31]
863_866del	Lys288IlefsTer77	10	fs	BOR	Orten et al., Hum. Mutat. (2008) [31]
866del	Asp289ValfsTer77	10	fs	BOR	Orten et al., Hum. Mutat. (2008) [31]
875dup	Asp293Ter	10	ns	BOR	Orten et al., Hum. Mutat. (2008) [31]
880C>T	Arg294Ter	10	ns	BOR	Kumar et al., Genet. Test. (1997) [40]
882del	Leu295CysfsTer71	10	fs	BOR	Krug et al., Hum. Mutat. (2011) [11]
889C>T	Arg297Ter	10	fs	BOR/BO	Rickard et al., J. Med. Gen. (2000); Wang et al., Zhonghua Er Bi Yan Hou Tou Jing Wai Ke Za Zhi (2020) [41,42]
920del	Arg307fsTer365	10	fs	BOR	Sanggaard et al., Eur. J. Hum. Genet. (2007) [43]
922C>T	Arg308Ter	10	ns	BOR/BO	Abdelhak et al., Nat. Gen. (1997); Orten et al., Hum. Mutat. (2008) [31,44]
965A>G	Glu322Gly	10	ms	BOR/BO	Song et al., PloS ONE (2013) [45]
966+5G>A	?	IVS10	sp	BOR/BO	Krug et al., Hum. Mutat. (2011); Stockley et al., Am. J. Med. Genet. A (2009) [11,23]
966_966+14del	splice junction loss	IVS10	fs	BOR	Krug et al., Hum. Mutat. (2011) [11]
967-1G>A	Invariant ‘ag’	IVS10	sp	BOR	Orten et al., Hum. Mutat. (2008) [31]
967-2A>G	Invariant ‘ag’	IVS10	sp	BOR	Kwon et al., Acta Otolaryngol. (2009) [46]
967A>T	Arg323	11	ns	BOR	Wang et al., BMC Med. Genet. (2018) [12]
977T>A	Ile326Asn	11	ms	BOR	Orten et al., Hum. Mutat. (2008) [31]
979T>C	Trp327Arg	11	ms	BO	Klingbeil et al., Int. J. Pediatr. Otorhinolaryngol. (2017) [35]
979T>G	Trp327Gly	11	ms	BOR	Masuda et al., Sci. Rep. (2022) [47]
989A>T	Glu330Val	11	ms	BOR	Krug et al., Hum. Mutat. (2011) [11]
1029del	Tyr344ThrfsTer22	11	fs	BOR	Orten et al., Hum. Mutat. (2008) [31]
1050+1G>T	Invariant ‘gt’	IVS11	sp	BOR	Orten et al., Hum. Mutat. (2008) [31]
1050+2T>C	Invariant ‘gt’	IVS11	sp	BOR	Unzaki et al., J. Hum. Genet. (2018) [24]
1050+3G>T	?	IVS11	sp	BOR	Masuda et al., Sci. Rep. (2022) [47]
1050+4A>C	exon skipping	IVS11	sp	BO	Chen et al., Clin. Exp. Otorhinolaryngol. (2023) [48]
1051-12T>G	New splice acceptor	IVS11	sp	BO	Orten et al., Hum. Mutat. (2008) [31]
1051-1G>C	Invariant ‘ag’	IVS11	sp	BOR	Okada et al., Pediatr. Nephrol. (2006) [49]
1054_1055insG	Pro352ArgfsTer26	12	fs	BOR	Masuda et al., Sci. Rep. (2022) [47]
1075_1077delinsAT	Gly359IlefsTer	12	fs	BO	Xing et al., Int. J. Pediatr. Otorhinolaryngol. (2020) [50]
1081C>T	Arg361Ter	12	ns	BOR/BO	Kumar et al., Genet. Test. (1997); Spruijt et al., Am. J. Med. Gen. A (2006) [40,51]
1088A>T	Glu363Val	12	ms	BOR	Krug et al., Hum. Mutat. (2011) [11]
1138G>T	Glu380Ter	12	ns	BOR	Krug et al., Hum. Mutat. (2011) [11]
1140+1G>A	?	IVS12	sp	BOR/BO	Song et al., PloS ONE (2013) [45]
1171del	Ser391fsTer9	12	fs	BOR	Lin et al., BMC Nephrol. (2023) [52]
1161_1164del	Ile387MetfsTer12	12	fs	BO	Unzaki et al., J. Hum. Genet. (2018) [24]
1118del	His373LeufsTer4	12	fs	BO	Orten et al., Hum. Mutat. (2008) [31]
1122del	Leu374PhefsTer6	12	fs	BOR	Unzaki et al., J. Hum. Genet. (2018) [24]
1138_1140+1del	Invariant ‘gt’	12; IVS12	sp	BOR	Orten et al., Hum. Mutat. (2008) [31]
1140+1G>A	Invariant ‘gt’	IVS12	sp	BOR/BO	Song et al., PloS ONE (2013) [45]
1141-1G>A	Invariant ‘ag’	13	fs	BOR	Sanggaard et al., Eur. J. Hum. Genet. (2007) [43]
1156del	His386IlefsTer2	13	fs	BO	Orten et al., Hum. Mutat. (2008) [31]
1189C>T	Gln397Ter	13	ns	BO	Ideura et al., Sci. Rep. (2019) [36]
1199+1G>C	Invariant ‘gt’	IVS13	sp	BOR	Krug et al., Hum. Mutat. (2011) [11]
1200-1G>A	Invariant ‘ag’	IVS13	sp	BO	Retterer et al., Genet. Med. (2016) [53]
1220G>A	Arg407Gln	14	ms	BO	Cho et al., Int. J. Mol. Sci. (2024) [8]
1254_1255del	Cys419PhefsTer32	14	fs	BO	Ideura et al., Sci. Rep. (2019) [36]
1255del	Cys419ValfsTer13	14	fs	BO	Ma et al., Zhonghua Er Bi Yan Hou Tou Jing Wai Ke Za Zhi (2021) [54]
1268del	Gly423ValfsTer9	14	fs	BO	Orten et al., Hum. Mutat. (2008) [31]
1276G>A	Gly426Ser	14	ms	BOR	Cho et al., Int. J. Mol. Sci. (2024) [8]
1286A>G	Asp429Gly	14	ms	BO	Namba et al., J. Hum. Genet. (2001); Yalcouyé et al., Mol. Genet. Genomic Med. (2022) [55,56]
1289G>A	Trp430Ter	14	ns	BOR	Unzaki et al., J. Hum. Genet. (2018) [24]
1315_1318dup	Arg440GlnfsTer13	14	fs	BOR	Krug et al., Hum. Mutat. (2011) [11]
1319G>A	Arg440Gln	14	ms	BOR	Unzaki et al., J. Hum. Genet. (2018) [24]
1329_1330	Glu443AspfsTer8	14	fs	BOR	Bałdyga et al., Genes (2023) [57]
1330_1331dup	Tyr445SerfsTer24	14	fs	BOR	Krug et al., Hum. Mutat. (2011) [11]
1350delinsCC	Asn451GlnfsTer10	14	fs	BO	Abdelhak et al., Nat. Genet. (1997) [44]
1360+4A>G	?	IVS14	sp	BOR	Sanggaard et al., Eur. J. Hum. Genet. (2007) [43]
1361-1G>A	Invariant ‘ag’	IVS14	sp	BOR	Riedhammer et al., Eur. J. Hum. Genet. (2023) [38]
1377_1378 delinsAT	Lys460Ter	15	ns	BOR	Orten et al., Hum. Mutat. (2008) [31]
1381del	Arg461GlyfsTer7	15	fs	BOR	Li et al., Intractable Rare Dis. Res. (2018) [58]
1405del	Ala469ProfsTer6	15	fs	BO	Orten et al., Hum. Mutat. (2008) [31]
1420_1421del	Leu474AspfsTer57	15	fs	BOR	Nardi et al., Clin. Nephrol. (2011) [59]
1471_1474dup	Arg492LeufsTer41	15	fs	BOR	Krug et al., Hum. Mutat. (2011) [11]
1475G>C	Arg492Pro	15	ms	BOR	Orten et al., Hum. Mutat. (2008) [31]
1475+1G>C	Invariant ‘gt’	15	sp	BOR	Gigante et al. BMC Nephrol.(2013) [60]
1476-2A>G	Invariant ‘ag’	IVS15	sp	BOR	Orten et al., Hum. Mutat. (2008) [31]
1487del	Val496GlyfsTer4	16	fs	BOR	Masuda et al., Sci. Rep. (2022) [47]
1493_1494insAT	Ile498PhefsTer3	16	fs	BOR	Chen et al., Int. J. Pediatr. Otorhinolaryngol. (2019) [61]
1496del	Leu499Ter	16	ns	BOR	Orten et al., Hum. Mutat. (2008) [31]
1510C>T	Gln504Ter	16	ns	BOR	Orten et al., Hum. Mutat. (2008) [31]
1524del	Leu509TrpfsTer9	16	fs	BOR	Krug et al., Hum. Mutat. (2011) [11]
1533dup	Val512SerfsTer20	16	fs	BOR	Krug et al., Hum. Mutat. (2011) [11]
1534G>T	Val512Phe	16	ms	BO	Orten et al., Hum. Mutat. (2008) [31]
1538T>C	Leu513Pro	16	ms	BO	Orten et al., Hum. Mutat. (2008) [31]
1541T>C	Leu514Pro	16	ms	BO/OTFC	Krug et al., Hum. Mutat. (2011); Mercer et al., Clin. Dysm. (2006) [11,15]
1570G>T	Glu524Ter	16	ns	BO	Orten et al., Hum. Mutat. (2008) [31]
1579T>A	Tyr527Asn	16	ms	BOR	Orten et al., Hum. Mutat. (2008) [31]
1580A>G	yr527Cys	16	ms	BOR	Orten et al., Hum. Mutat. (2008) [31]
1591A>T	Lys531Ter	16	ns	BOR	Orten et al., Hum. Mutat. (2008) [31]
1597G>A	Gly533Arg	16	ms	BO	Castiglione et al., Int. J. Pediatr. Otorhinolaryngol. (2014) [6]
1597+1G>A	Invariant ‘gt’	IVS16	sp	BOR	Tian et al., Prenat. Diagn. (2024) [62]
1598-2A>C	Invariant ‘at’	IVS16	sp	BOR/BO	Song et al., PloS ONE (2013) [45]
1603_1607del	Glu535LeufsTer3	17	fs	BOR	Orten et al., Hum. Mutat. (2008) [31]
1623_1626dup	Gln543AsnfsTer90	17	fs	BOR	Cho et al., Int. J. Mol. Sci. (2024) [8]
1627C>T	Gln543Ter	17	ns	BOR	Spahiu et al., Balkan J. Med. Genet. (2016) [63]
1644del	Val549TrpfsTer6	17	fs	BO	Orten et al., Hum. Mutat. (2008) [31]
1641_1645del	Arg547SerfsTer83	17	fs	BOR	Krug et al., Hum. Mutat. (2011) [11]
1643_1644dup	Val549LysfsTer7	17	fs	BOR	Unzaki et al., J. Hum. Genet. (2018) [24]
1649T>A	Val550Glu	17	ms	BO	Orten et al., Hum. Mutat. (2008) [31]
1653T>G	Tyr551Ter	17	ns	BOR	Krug et al., Hum. Mutat. (2011) [11]
1657_1659del	Val553del	17	indel	BOR	Orten et al., Hum. Mutat. (2008) [31]
1697dup	His567AlafsTer65	17	fs	BOR	Orten et al., Hum. Mutat. (2008) [31]
1697_1698delAGinsT	Lys566IlefsTer73	17	fs	BO	He et al., Front. Genet. (2024) [64]
1698+1G>T	Invariant ‘gt’	17	sp	BOR	Orten et al., Hum. Mutat. (2008) [31]
1706T>C	Met569Thr	18	ms	BO	Krug et al., Hum. Mutat. (2011) [11]
1715G>T	Trp572Leu	18	ms	BO	Feng et al., Zhonghua Er Bi Yan Hou Tou Jing Wai Ke Za Zhi (2022) [65]
1715G>A	Trp572Ter	18	ns	BOR	Cho et al., Int. J. Mol. Sci. (2024) [8]
1716G>A	Trp572Ter	18	ns	BO	Orten et al., Hum. Mutat. (2008) [31]
1730_1745del	His577ProfsTer57	18	fs	BO	Unzaki et al., J. Hum. Genet. (2018) [24]
1735del	Asp579ThrfsTer60	18	fs	BOR	Wang et al., Laryngoscope (2012) [34]
1744del	Ala582ProfsTer57	18	fs	BO	Shao et al., Lin Chuang Er Bi Yan Hou Tou Jing Wai Ke Za Zhi (2024) [66]
1754dup	His585GlnfsTer47	18	fs	BOR	Krug et al., Hum. Mutat. (2011) [11]
1766dup	Glu590GlyfsTer42	18	fs	BOR	Masuda et al., Sci. Rep. (2022) [47]
1768del	Glu590SerfsTer49	18	fs	BO	Klingbeil et al., Int. J. Pediatr. Otorhinolaryngol. (2017) [35]
1773C>G	Tyr591Ter	18	ns	BO	Sanggaard et al., Eur. J. Hum. Genet. (2007) [43]
1777T>A	Ter593LysextTer6	18	sl	BO	Krug et al., Hum. Mutat. (2011) [11]
1777_1778delTAinsGT	Ter593Val	18	sl	BO	Matsunaga et al., Acta Otolaryngol. (2007) [67]

?: unknown consequences.

**Table 4:** Reported patients with otofaciocervical syndrome (OTFCS) carrying *EYA1* (NM_000503.6) variants, compared with the current case. Clinical features are grouped into core domains: HL = hearing loss; BA = branchial anomalies; EA = external ear anomalies; RA = renal anomalies; MSK = musculoskeletal anomalies; NDD = neurodevelopmental delay; ST = short stature. Additional findings are listed under “Other”. Variants are described according to NM_000503.6 (*EYA1*) and mapped to the GRCh38 assembly. Variant type was classified as single-nucleotide variant (SNV) or copy-number variant (CNV). Inheritance is indicated when available.

Reference	Patients (n.)	HL	BA	EA	RA	MSK	NDD	ST	Other	Genotype	Variant Type	Inheritance
Vincent et al., 1994 [68]	1	+	+	NT	+	+	+	−	Hydrocephalus	8q12.2–q21.2del	CNV	de novo
Rickard et al., 2001 [14]	1	+	+	+	+	+	+	+	−	del(ex7,9,13)	CNV	de novo
	2	+	+	+	+	−	+	−	−	del(ex7,9,13)	CNV	de novo
Estefanía et al., 2006 [13]	1	+	+	+	+	+	−	−	IgA deficiency	c.639+1G>A	SNV	de novo
Mercer et al., 2006 [15]	1	+	+	+	+	+	+	+	−	c.1442T>C	SNV	NT
This study	1	+	+	+	−	+	+	+	−	8q13.2q13.3del	CNV	de novo

HL, hearing loss; BA, branchial anomalies; EA, ear anomalies; RA, renal anomalies; MSK, musculoskeletal anomalies; NDD, neurodevelopmental delay; ST, short stature; NT, not tested; CNV, copy number variant; SNV, single nucleotide variant. +, present; −, absent.

## Data Availability

All datasets used and/or analyzed during the current study are available from the corresponding author upon reasonable request.

## Supplementary Materials

The following supporting information can be downloaded at: https://www.mdpi.com/article/10.3390/genes16111267/s1, Supplementary File: Flow diagram for literature review and data extraction via databases and registers.
