# Supplementary material for: Otofaciocervical Syndrome and Its Overlap with Branchiootorenal Spectrum: An Integrated Literature Analysis of EYA1-Related Disorders, Including a Novel Case with an 8q13.2q13.3 Deletion
Source: Genes (Basel). 2025 Oct 28;16(11):1267. doi: 10.3390/genes16111267 (PMC12652286; doi:10.3390/genes16111267)
Supplement: Supplementary file 1 [file genes-16-01267-s001.zip › genes-3873757-supplementary.pdf]

Flow diagram for literature review and data extraction

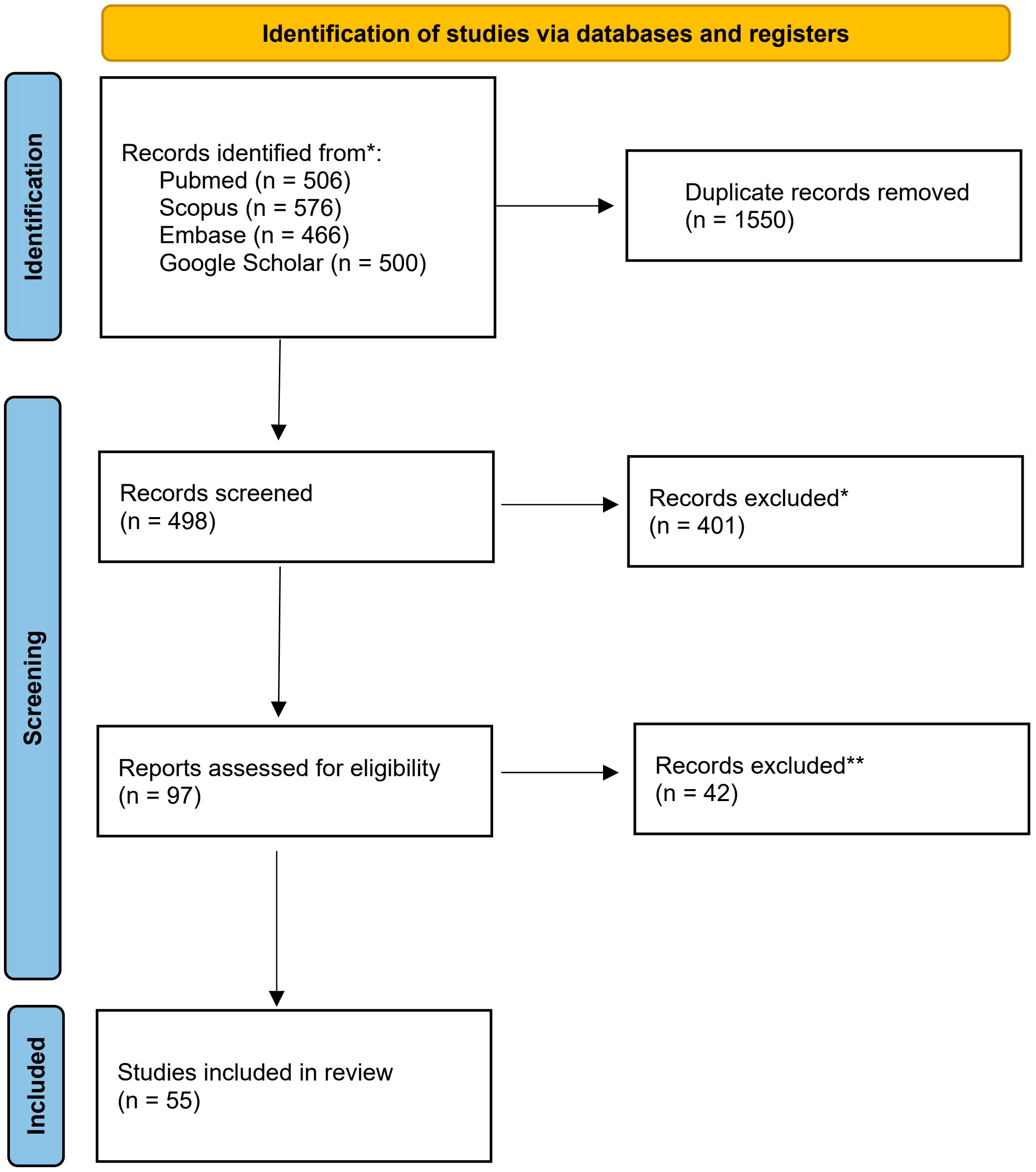

* Title, Abstract, Diagonal reading.

**Absent genetic information and/or patient-level clinical data covering less than 2 domains (branchial, otologic, renal, craniofacial, musculoskeletal) and/or overlapping cohorts and/or non-human models only and/or *EYA1* complex rearrangements.
